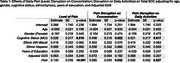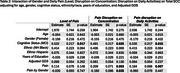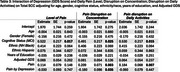# Elucidating the Association Between Pain and Self‐Perceived Cognitive Functioning Using Daily Digital Diary Assessments: Results from the Einstein Aging Study (EAS)

**DOI:** 10.1002/alz70857_107072

**Published:** 2025-12-25

**Authors:** Angel Garcia De La Garza, Carol A. Derby, Cuiling Wang, Nelson A. Roque, Mindy J. Katz, Richard B. Lipton, Laura A. Rabin

**Affiliations:** ^1^ Albert Einstein College of Medicine, Bronx, NY, USA; ^2^ The Pennsylvania State University, University Park, PA, USA; ^3^ Brooklyn College of the City University of New York, Brooklyn, NY, USA; ^4^ The Graduate Center, CUNY, New York, NY, USA

## Abstract

**Background:**

Pain is a common symptom among older adults and has been linked to cognitive concerns, yet its day‐to‐day impact on subjective cognitive functioning remains unclear. Using smartphone‐based ecological momentary assessments (EMA), we examine associations between daily self reports of pain and daily reports of subjective cognitive concerns (SCC) in community‐dwelling older adults. EMA approaches provide real‐time data, reduces recall bias and captures within‐person fluctuations in pain and cognition. These dynamic relationships can inform how transient changes in pain influence cognitive self‐perceptions and daily functioning.

**Methods:**

Participants from the EAS completed six daily survey assessments over 14 days. Cognitive lapses in everyday functioning were assessed once per day in the night survey, where participants also rated their pain level throughout the entire day (0–100). Across all six surveys each day, they reported the extent to which pain interfered with daily activities and their ability to concentrate. We calculated daily averages for pain interference in daily activities and concentration. Linear mixed‐effects models examined whether pain reports were associated with the number total number of SCC reported on a given day, adjusting for age, sex, race/ethnicity, depression status, and cognitive status (unimpaired or mild cognitive impairment). We also explored interactions between pain and demographic or clinical factors, including sex, race, depression (GDS scale), and age.

**Results:**

Analyses included 310 community‐dwelling participants (mean age = 77.5, SD = 4.94; 66.4% female; 47.1% non‐Hispanic White, 40.7% non‐Hispanic Black, 12.3% Hispanic; 70% cognitively unimpaired). Daily pain level (*p* < 0.001), average pain interference in daily activities (*p* = 0.032), and average pain interference in concentration (*p* = 0.027) were associated with a higher reports of SCCs. Significant interactions emerged between gender, depression, and pain. Females showed weaker associations between the day average of pain interferrence on concentration and total SCCs (*p* = 0.035). Additionally, participants with higher GDS scores exhibited greater effects of pain levels on total reported SCCs (*p* = 0.035). No significant interactions were found between cognitive status, age, or ethnicity.

**Conclusions:**

Results indicate that daily experiences of pain may impact self‐perceived cognitive functioning. Findings suggest that pain management is important to preserving cognitive health.